# Evaluation of the nutritional status in patients with COVID-19

**DOI:** 10.3164/jcbn.20-91

**Published:** 2020-08-06

**Authors:** Chenchen Wei, Ya Liu, Yu Li, Yi Zhang, Ming Zhong, Xiao Meng

**Affiliations:** 1 The Key Laboratory of Cardiovascular Remodeling and Function Research, Chinese Ministry of Education, Chinese National Health Commission and Chinese Academy of Medical Sciences, The State and Shandong Province Joint Key Laboratory of Translational Cardiovascular Medicine, Department of Cardiology, Qilu Hospital, Cheeloo College of Medicine, Shandong University, No. 107, Wenhua xi road, Jinan 250012, China; 2 Department of Gastroenterology, Renmin Hospital of Wuhan University, Gaoxin 6th Road, Donghu High Tech Development Zone, Wuhan 430060, China; 3 Department of Respiratory Medicine, Qilu Hospital, Cheeloo College of Medicine, Shandong University, No. 107, Wenhua xi road, Jinan 250012, China

**Keywords:** 2019-nCoV, COVID-19, malnutrition, the CONUT score, prognosis

## Abstract

COVID-19 has been a global health threat. We aimed to investigate the nutrition status of COVID-19 patients and evaluate the prognostic value of the controlling nutritional status (CONUT) score in these patients. 348 severe patients with COVID-19 were collected. Based on the CONUT score, 161 (46.3%) patients had mild malnutrition while 139 (39.9%) patients had moderate-severe malnutrition. Compared to the patients in normal and mild groups, the patients in moderate-severe group were older, more male, had higher counts of white blood cell and neutrophil as well as higher serum levels of C-reactive protein. Nearly half of patients (44.6%) in moderate-severe group developed acute cardiac injury, while 6.3% and 15.5% patients in normal and mild group, respectively. Patients with moderate-severe malnutrition exhibited a higher mortality than those patients with normal and mild malnutrition. Multivariate regression analysis showed the CONUT score was the independent predictor of death in patients with COVID-19 (odds ratio: 1.410; 95%CI: 1.089–1.825; *p* = 0.009). Malnutrition is significantly associated with poor outcome of COVID-19, while the prognosis of patients with normal nutrition status is relative favorable. The CONUT score independently predicts the prognosis of COVID-19 patients, which can help physicians to clarify patients with poor prognosis.

## Introduction

A novel coronavirus, named 2019-nCoV by the World Health Organization (WHO) was first found in December 2019 in China, causing an outbreak of pneumonia (COVID-19) in the following month. It is human-to-human transmission and rapidly transmitted. Although most patients present mild symptoms and quickly recover, the disease can cause severe respiratory disease, multiple organ dysfunction and even death.^(1)^ Nowadays, COVID-19 has spread to many other countries around the world, including Italy, Spain, Iran, South Korea, Japan, and the United States. 3,024,059 confirmed cases have been reported worldwide by WHO as of April 30, 2020, including 208,112 deaths. Now, it has been a global health threat. Unfortunately, there is still no specific therapeutic and preventive treatment.

As the nutritional status of the host exerts a crucial role in the defense against infection, individuals with nutritional deficiency are more susceptible to a series of infectious disease which can result a detrimental consequence.^(2,3)^ Malnutrition has been considered as an independent risk factor for increased complications and higher mortality in hospitalized patients.^(4)^ The basic nutritional status of elderly patients with chronic diseases is always poor, which makes them tend to be critically ill after infection.^(1)^ Therefore, it is important to evaluate nutritional status of the patients accurately.

The controlling nutritional status (CONUT) score is a novel comprehensive index, which is calculated from lymphocyte counts, total cholesterol (TC) and serum albumin levels.^(5)^ As a simple, economic and efficient screening tool to detect the nutritional status of inpatients without requiring extra skills, the CONUT score has been applied in many tumors for its prognostic value.^(6,7)^ Recently, it was reported that the CONUT score was independently associated with the poor prognosis in many cardiovascular disease.^(8,9)^ Recently, Restrepo and colleagues proposed a view that “pneumonia as a cardiovascular disease”.^(10)^ Therefore, whether there is an association between the CONUT score and COVID-19 should be determined.

To our best knowledge, almost all studies have focused on the description of the epidemiology and clinical characteristics of patients with COVID-19 while there is nearly no study to assess the nutritional status in these patients. So, we aimed to investigate the clinical impact of nutritional screening in patients with COVID-19 and evaluate the prognostic value of the CONUT score in these patients.

## Materials and Methods

### Patients

In this retrospective and single-center study, we recruited 348 consecutive severe patients with confirmed COVID-19 who were hospitalized from January 3, 2020 to March 11, 2020, at Renmin Hospital of Wuhan University. The patients were all severe cases and diagnosed according to the guidelines issued by the China National Health Commission, which met at least one of the following criteria: (1) symptoms of tachypnea with respiratory rate ≥30 times/min; (2) the saturation level of blood oxygen under resting state decreased to ≤93%; (3) oxygenation index (PaO_2_/FiO_2_) ≤300 mmHg (1 mmHg = 0.133 kPa); (4) lung infiltrates more than 50% within 24 to 48 h.^(11)^ The study was approved by the Ethics Committees of Qilu Hospital of Shandong University and Renmin Hospital of Wuhan University.

### Data collection

Information of enrolled patients was obtained through the electronic medical record system, including epidemiological, clinical and laboratory characteristics, treatment options and outcomes. The CONUT score was calculated on admission. As previous described,^(5)^ the CONUT score was calculated from lymphocyte counts, TC and serum albumin levels (Supplemental Table 1*). Based on the CONUT score, patients were categorized into three groups: normal nutritional status (CONUT 0–1), mild malnutrition (CONUT 2–4), and moderate-severe malnutrition (CONUT ≥5). Higher score suggested a worse undernutrition of the patients. Acute cardiac injury was defined as serum cardiac troponin I levels (cTnI) were above the upper limit of the reference range (>0.04 ng/ml).

### Statistical analyses

All data were statistically analyzed using SPSS statistics for Windows 25.0 (SPSS Inc. Chicago, IL). We assessed the normal distribution of continuous variables by the Shapiro-Wilk test before statistical analysis. Non-normally distributed variables were presented as median (IQR) and the Mann-Whitney *U* test and Kruskal-Wallis ANOVA were used for comparisons between groups. Categorical variables were expressed as number (percentage) and were compared using the χ^2^ test or Fisher’s exact test. Univariate logistic regression analysis and multivariate logistic regression analysis were performed to determine Independent prognostic factors. Only those variables that were identified as significant in the univariate analysis were included in the multivariate analysis. With the CONUT score as test variable and prognosis as state variable, receiver operating characteristic (ROC) curve was formulated and the diagnostic value of the CONUT score was evaluated based on area under the curve (AUC). The optimal cut-off value was determined using the Youden index. All of the statistical tests were two-sided, and *p*<0.05 was considered to be statistically significant.

## Results

### Presenting characteristics

A total of 348 patients meeting the criteria for severe COVID-19 were included in this study. Among them, the median age was 66 years (IQR 56–73 years), including 47.7% female and 52.3% male. There was no case in children and adolescence while over half of cases were 65 years of age or older. Many patients also suffered from underlying disease, including hypertension (42.2%), diabetes (17.5%), coronary artery disease (CAD, 13.5%), cancer (2.9%), chronic kidney disease (CKD, 2.6%), and chronic obstructive pulmonary disease (COPD, 1.7%). Most patients had fever (84.8%) and cough (62.9%). Others common symptoms included fatigue (48.9%), short of breath (30.5%), dyspnea (16.4%), and diarrhea (15.5%).

Based on the system of CONUT score, 48 (13.8%) patients had normal nutritional status, 161 (46.3%) patients had mild malnutrition, and 139 (39.9%) patients had moderate-severe malnutrition. There was no significant difference in symptoms, heart rate and blood pressure among the three groups of patients (Table 1).

### Laboratory findings

Compared to patients with normal and mild malnutrition, patients in moderate-severe group were older and more male (*p*<0.05). The patients with moderate-severe malnutrition suffered from more hypertension and diabetes. Compared to the patients in normal and mild groups, the counts of white blood cell and neutrophil and the serum levels of C-reactive protein (CRP), aspartate aminotransferase (AST), lactate dehydrogenase (LDH), creatine kinase-myocardial isoenzyme (CK-MB) were higher, while lymphocytes, platelet, albumin, and TC values were lower in moderate-severe group (*p*<0.05). According to the serum levels of cTnI, nearly half of patients (44.6%) in moderate-severe group developed acute cardiac injury, while 6.3% and 15.5% patients in normal and mild group, respectively (*p*<0.05). Importantly, patients with moderate-severe malnutrition exhibited a lower survival to discharge and a higher mortality than those with normal and mild malnutrition (50.4% vs 100.0% vs 82.0%; 43.2% vs 0.0% vs 13.0%, respectively, *p*<0.05, Table 1).

### The comparison of CONUT score between survivors and non-survivors

Until March 11, 2020, 250 (71.8%) patients discharged while 81 (23.3%) patients died. In addition, 17 (4.9%) patients were still hospitalized. After excluding these 17 inpatients, 331 patients were divided into two groups: survival and non-survival groups. For survivors, the median of CONUT score was 3.0 (IQR 2.0–5.0) while 6.0 (IQR 4.0–7.0) in non-survivors (*p*<0.05). In subgroup analysis based on the counts of lymphocytes (≥0.8 × 10^9^/L), we found the median of CONUT score was 3.0 in 196 survivors, while 4.0 in 27 non-survivors. When the lymphocytes were less than 0.8 × 10^9^/L, the CONUT score was 5.0 in 54 survivors while 6.0 in 54 non-survivors. The results indicated that the CONUT score is significantly higher in non-survivors than those in survivors regardless of the counts of lymphocyte. (*p*<0.05, Table 2).

### The CONUT score predicts the prognosis of patients with COVID-19

The data of 331 patients (survivors and non-survivors) were analyzed in the univariate and multivariable logistic regression model. The multivariate logistic regression analysis determined that the CONUT score remained an independent predictor of all-cause mortality even after adjusting the model for multiple covariates (odds ratio: 1.410; 95%CI: 1.089–1.825; *p* = 0.009). In addition, we found that advanced age, dyspnea, CAD, higher serum levels of CRP and LDH levels, and acute cardiac injury were also independently associated with increased mortality (Table 3).

**Fig. 1:**
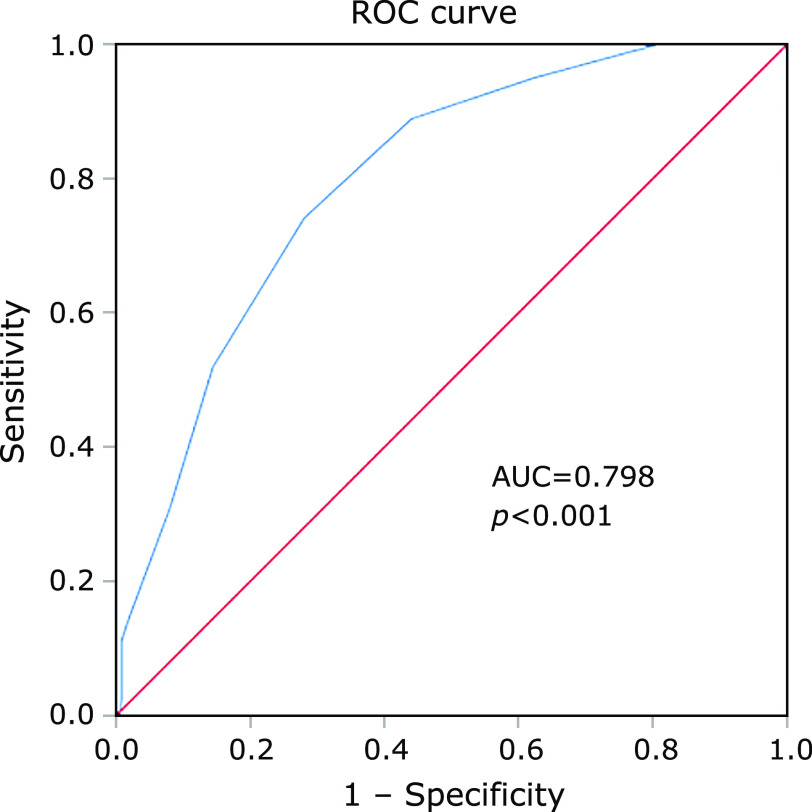
The receiver operating characteristic (ROC) analysis was used to evaluate the diagnostic value of CONUT score and estimate the optimal cut-off value. AUC, area under curve.

ROC analysis was also used to assess the power of CONUT score in the prognosis of patients with COVID-19. The CONUT score and prognosis of patients were used as the test variable or the state variable, respectively. The area under the ROC curve was 0.798 (95%CI: 0.847–0.956, *p*<0 .001). The large area under ROC curve shows the comparatively high predictive value of prognosis of COVID-19 patients based on the CONUT score. For all of the COVID-19 patients, the CONUT score  =  4.5 had a maximizing the Youden index, with a sensitivity and specificity of 74.1% and 72.0%, respectively (Fig. 1).

## Discussion

Nutritional status can influence viral genome mutations from a benign or mildly pathogenic virus to a highly virulent one and its spread in hosts.^(3)^ Therefore, evaluating the nutritional status of patients precisely is helpful for clinicians to identify the disease progression and apply therapy strategy. However, the assessment of nutritional status of subjects has not been regarded as a beneficial factor to viral infectious diseases and always ignored clinically. There were four main findings in our present study. Firstly, inflammatory indicators and acute cardiac injury were significantly higher in patients combined with COVID-19 and moderate-severe malnutrition; Secondly, patients in moderate-severe group had a lower survival to discharge and a higher mortality than those in normal and mild groups; Thirdly, non-survivors had a higher CONUT score than survivors in patients with COVID-19, suggesting they had a worse nutrition state; Fourthly, the CONUT score was an independent predictor for mortality in patients with COVID-19. To our best knowledge, this study is the first to reveal the relationship between the nutritional status and the prognosis in patients with COVID-19.

The CONUT score can be easily calculated for evaluation the nutritional status of inpatients and without requiring the extra anthropometric parameters.^(5)^ In the present study, 348 severe cases were enrolled and their nutritional status were evaluated by CONUT score. We found the patients combined COVID-19 and moderate-severe malnutrition were older, more male and had more comorbidities. Therefore, the patients with an elderly age or having chronic diseases should receive a nutrition screening.

Some 2019-nCoV-infected patients progress rapidly, accompanied with acute respiratory distress syndrome (ARDS), multiple organ failure and even death. One study enrolling 41 patients showed that 15% patients died and the mortality rate was 38% in patients requiring ICU admission.^(12)^ Recently, another study enrolling 52 critically ill adult patients with COVID-19 reported that 32 (61.5%) patients died at 28 days.^(13)^ This heterogeneity of the results may be due to the difference in severity of enrolled patients. In this study, we enrolled 348 severe cases and found the mortality was 23.3%. In the patients combined with COVID-19 and moderate-severe malnutrition, the mortality increased to 43.2%, which was higher than that in patients with normal and mild malnutrition. Meanwhile, the patients with moderate-severe malnutrition showed a lower discharge rate. Therefore, poor nutritional status may contribute to an increased incidence of death in COVID-19 patients.

Virus invasion is able to result in the changes of white blood cells in peripheral blood, induce a cytokine storm and thereby generate a series of immune response.^(1,14)^ White blood cell and neutrophil counts were related to cytokine storms caused by virus invasion. 2019-nCoV infection can cause exuberant inflammatory response and uncontrolled pulmonary inflammation may be the major cause of fatality in COVID-19.^(15)^ Most of patients with COVID-19 exhibited elevated CRP levels which was more prominent in severe cases than non-severe cases.^(1,16)^ Inflammation and malnutrition always exist concomitantly as malnutrition can enhance the susceptibility to infections; Meanwhile, infections further promote malnutrition via increased demand for nutrients and decreased appetite.^(17)^ In one study by Eckart *et al.*^(18)^ enrolling 2,465 patients, elevated serum CRP concentration was related to low albumin levels, suggesting increased elevated inflammatory parameters was independently associated with hypoalbuminemia. In this study, the patients with moderate-severe malnutrition had higher counts of white blood cell and neutrophil and elevated serum levels of CRP, suggesting an active inflammatory state.

Although respiratory failure has been considered as a leading cause of death in COVID-19 patients, acute cardiac injury is also one of the important causes of this disease. Virus, inflammation and hypoxia state can directly trigger cardiac damage, and thereby some patients may die of fulminant myocarditis.^(15)^ In one study by Ruan *et al.*,^(15)^ 36 patients (53%) died of respiratory failure while 40% of deaths were associated with circulatory failure due to cardiac injury among the 68 fatal cases. Shi *et al.*^(19)^ reported that among 416 patients with COVID-19, 82 (19.7%) patients had cardiac injury, who exhibited a higher in-hospital mortality than those without cardiac injury (51.2% vs 4.5%). They indicated that cardiac injury was a common condition among the COVID-19 patients and related to higher risk of mortality.^(19)^ In our study, we found the patients with moderate-severe malnutrition had significantly higher serum levels of AST, LDH, and CK-MB. Moreover, elevated serum cTnI level was observed in nearly half of patients in moderate-severe group, suggesting that patients combined with COVID-19 and malnutrition were more likely to suffer from acute cardiac injury. This may be one of the contributing causes to the high in-hospital mortality in these patients combined with COVID-19 and malnutrition.

Immune cells exert a central role in effective host response to various pathogens while deficiency of immune cells disrupts immune homeostasis, causing pathological conditions. Recently Qin *et al.*,^(20)^ reported that dysregulation of immune system involved in the pathological process of COVID-19. The patients with weaker immune functions were more likely infected by 2019-nCoV.^(1)^ As nutrition and immunity are closely linked, inadequate nutrition may lead to weakened immune function.^(3)^ Peripheral lymphocyte count is an indicator of immunological and nutritional status. Many infected patients experienced lymphopenia in which the decrease of primary immune cell counts was related to the severity of disease.^(12–14)^ It commended that in patients with COVID-19, more attention should be paid to lymphopenia or an obviously decrease in the number of CD4 and CD8 T cells.^(21)^ In our study, lower counts of lymphocyte and CD4 and CD8 T cell were observed in patients with moderate-severe malnutrition, indicating a damaged immune function. In order to exclude the bias caused by lymphopenia, subgroup analysis was performed. Regardless of the counts of lymphocyte, the CONUT score remained significantly higher in non-survivors than those in survivors.

In order to clarify the independent predictors of death in patients with COVID-19, we performed multivariate regression analysis and found that the CONUT score was a significant and independent predictor for death in these patients. In addition, advanced age, comorbidity, elevated inflammatory indicators and acute cardiac injury were also independent associated with increased mortality, which was consistent with previous studies.^(15)^ The ROC curves also emphasized the predictive value of CONUT score in the prognosis of the patients with COVID-19, with a higher sensitivity and specificity.

There were several limitations in this study. First, this was a retrospective and single-center study. Thus, it may lead to biases in patient selection. Second, the CONUT score might have been biased by medicine or the presence of undetected conditions. A prospective study should be performed to overcome these limitations.

In conclusion, patients combined with COVID-19 and malnutrition exhibited elevated inflammatory response, more acute cardiac injury and weakened immune function. Malnutrition is significantly associated with poor outcome of COVID-19, while the prognosis of patients with normal nutrition status is relatively favorable. Therefore, nutritional status of the patients with COVID-19 should be paid particular attention and carefully evaluated at early stage. As a convenient and effective method, the CONUT score can independently predict the prognosis of COVID-19 patients. So, CONUT score can help physicians to clarify patients with poor prognosis and provide individualized treatment to improve their survival.

## Author Contributions

CW, YL, YL, and YZ collected the epidemiological and clinical data and performed the statistical data. CW and XM drafted the manuscript. MZ designed the study. MZ and XM are responsible for summarizing all data. All authors read and approved the final manuscript.

## Figures and Tables

**Table 1:** Demographics characteristics, laboratory findings and clinical outcome of patients with COVID-19

	CONUT score			
	All Patients (*n* = 348)	Normal (*n* = 48)	Mild malnutrition (*n* = 161)	Moderate-severe malnutrition (*n* = 139)
Age (years)	66.0 (56.0–73.0)	63.0 (52.8–67.0)	64.0 (52.0–70.0)	70.0 (63.0–77.0)*^,^^†^
Gender				
Male	182 (52.3%)	13 (27.1%)	82 (50.9%)*	87 (62.6%)*^,^^†^
Female	166 (47.7%)	35 (72.9%)	79 (49.1%)*	52 (37.4%)*^,^^†^
Signs and symptoms				
Fever	295 (84.8%)	36 (75.0%)	138 (85.7%)	121 (87.1%)
Dry cough	219 (62.9%)	32 (66.7%)	98 (60.9%)	89 (64.0%)
Fatigue	170 (48.9%)	23 (47.9%)	75 (46.6%)	72 (51.8%)
Short of breath	106 (30.5%)	13 (27.1%)	41 (25.5%)	52 (37.4%)
Dyspnea	57 (16.4%)	4 (8.3%)	26 (16.1%)	27 (19.4%)
Diarrhea	54 (15.5%)	4 (8.3%)	29 (18.0%)	21 (15.1%)
Heart rate (bpm)	85.0 (77.0–96.8)	82.5 (74.5–97.3)	86.0 (78.0–97.5)	84.0 (77.0–96.0)
SBP (mmHg)	128.0 (119.0–142.0)	124.0 (119.0–141.5)	127.0 (118.5–139.5)	130.0 (119.0–145.0)
DBP (mmHg)	78.0 (70.0–85.0)	78.0 (71.0–84.5)	78.0 (70.0–85.0)	77.0 (70.0–85.0)
Comorbidity				
Hypertension	147 (42.2%)	21 (43.8%)	57 (35.4%)	69 (49.6%)^†^
Diabetes	61 (17.5%)	2 (4.2%)	26 (16.1%)*	33 (23.7%)*
CAD	47 (13.5%)	5 (10.4%)	19 (11.8%)	23 (16.5%)
Cancer	10 (2.9%)	0 (0.0%)	5 (3.1%)	5 (3.6%)
CKD	9 (2.6%)	0 (0.0%)	4 (2.5%)	5 (3.6%)
COPD	6 (1.7%)	1 (2.1%)	1 (0.6%)	4 (2.9%)
Laboratory findings				
White blood cells (10^9^/L)	5.71 (4.33–7.81)	6.40 (4.97–7.57)	5.29 (4.17–6.71)	6.51 (4.40–9.13)^†^
Neutrophils (10^9^/L)	3.98 (2.71–6.06)	3.69 (2.38–5.09)	3.43 (2.38–4.97)	5.15 (3.22–8.09)*^,^^†^
Lymphocytes (10^9^/L)	1.01 (0.68–1.47)	1.84 (1.61–2.12)	1.21 (0.88–1.49)*	0.68 (0.45–0.92)*^,^^†^
Hemoglobin (g/L)	122.0 (111.0–134.0)	123.0 (116.0–132.0)	124.0 (113.0–136.5)	121.0 (106.0–131.0)
Platelet (10^9^/L)	210.5 (156.3–274.5)	252.5 (203.3–371.3)	222.0 (168.5–284.5)*	178.0 (138.0–249.0)*^,^^†^
CRP (mg/L)	42.3 (5.5–77.7)	5.0 (0.0–28.2)	25.0 (2.6–55.6)*	65.5 (37.4–111.5)*^,^^†^
ALT (U/L)	24.0 (16.0–38.0)	23.0 (14.3–35.0)	23.0 (15.5–39.0)	26.0 (17.0–39.0)
AST (U/L)	29.0 (19.0–42.0)	21.5 (18.0–34.8)	26.0 (19.0–37.5)	33.0 (23.0–50.0)*^,^^†^
Albumin (g/L)	36.5 (33.1–39.3)	39.8 (37.6–42.2)	37.8 (36.1–40.3)*	33.0 (31.1–34.6)*^,^^†^
Creatinine (µmol/L)	61.0 (50.0–79.8)	56.0 (47.5–68.0)	60.0 (50.0–74.0)	67.0 (53.0–90.0)*^,^^†^
Total bilirubin (µmol/L)	10.85 (8.20–15.18)	10.55 (7.95–14.25)	10.10 (7.95–14.75)	11.60 (8.40–16.20)
Blood glucose (mmol/L)	5.81 (5.02–7.46)	5.25 (4.76–6.17)	5.53 (4.88–7.10)	6.78 (5.47–8.79)*^,^^†^
TC (mmol/L)	3.83 (3.32–4.37)	4.80 (4.21–5.64)	3.93 (3.43–4.50)*	3.46 (3.00–3.94)*^,^^†^
HDL-C (mmol/L)	0.91 (0.74–1.11)	1.18 (0.94–1.41)	0.95 (0.81–1.12)*	0.79 (0.66–0.98)*^,^^†^
LDL-C (mmol/L)	2.39 (1.85–2.88)	3.09 (2.61–3.61)	2.46 (2.02–2.95)*	2.07 (1.63–2.53)*^,^^†^
TG (mmol/L)	1.27 (0.96–1.68)	1.55 (1.12–2.52)	1.25 (0.95–1.64)*	1.24 (0.95–1.60)*
Potassium (mmol/L)	3.98 (3.57–4.40)	4.11 (3.76–4.43)	3.96 (3.61–4.36)	3.91 (3.49–4.38)
Sodium (mmol/L)	141.0 (138.0–145.0)	144.0 (141.0–146.8)	141.0 (139.0–144.0)*	141.0 (137.0–145.0)*
LDH (U/L)	276.0 (208.8–405.3)	204.0 (182.8–284.0)	249.0 (199.5–321.0)	353.0 (271.0–493.0)*^,^^†^
CK-MB (ng/ml)	1.21 (0.75–2.69)	1.05 (0.66–1.49)	1.03 (0.72–2.09)	2.01 (0.98–3.41)*^,^^†^
Acute cardiac injury	90 (25.9%)	3 (6.3%)	25 (15.5%)	62 (44.6%)*^,^^†^
CD4 (/µl)	419.0 (241.0–556.5)	730.5 (573.3–909.0)	430.2 (308.5–560.5)*	277.0 (151.0–430.2)*^,^^†^
CD8 (/µl)	220.5 (118.5–317.8)	412.5 (263.5–486.0)	237.8 (146.5–334.5)*	149.0 (67.0–237.8)*^,^^†^
Treatment				
Antiviral therapy	341 (98.0%)	47 (97.9%)	158 (98.1%)	136 (97.8%)
Antibiotic therapy	276 (79.3%)	31 (64.6%)	125 (77.6%)	120 (86.3%)*
Glucocorticoids	137 (39.4%)	12 (25.0%)	51 (31.7%)	74 (53.2%)*^,^^†^
Clinical outcome				
Remained in hospital	17 (4.9%)	0 (0.0%)	8 (5.0%)	9 (6.5%)
Discharge	250 (71.8%)	48 (100.0%)	132 (82.0%)*	70 (50.4%)*^,^^†^
Death	81 (23.3%)	0 (0.0%)	21 (13.0%)*	60 (43.2%)*^,^^†^

Data were presented as *n* (%) or median (IQR). *p<0.05 vs normal group; ^†^*p*<0.05 vs mild malnutrition group. SBP, systolic blood pressure; DBP, diastolic blood pressure; CAD, coronary artery disease; CKD, chronic kidney disease; COPD, chronic obstructive pulmonary disease; CRP, C-reactive protein; ALT, alanine aminotransferase; AST, aspartate aminotransferase; TC, total cholesterol; HDL-C, high density lipoprotein cholesterol; LDL-C, low density lipoprotein cholesterol; TG, triglyceride; LDH, lactate dehydrogenase; CK-MB, creatine kinase-myocardial isoenzyme.

**Table 2:** The comparison of CONUT score between survivors and non-survivors

		Survival (*n* = 250)	non-survival (*n* = 81)	*p*
All Patients	*n*	250	81	
	CONUT score	3.0 (2.0–5.0)	6.0 (4.0–7.0)	0.000
Lymphocyte counts ≥800 (/mm^3^)	*n*	196 (78.4%)	27 (33.3%)	
	CONUT score	3.0 (2.0–4.0)	4.0 (3.0–5.0)	0.000
Lymphocyte counts <800 (/mm^3^)	*n*	54 (21.6%)	54 (66.7%)	
	CONUT score	5.0 (4.0–7.0)	6.0 (5.0–7.0)	0.002

Data were presented as *n* or median (IQR). CONUT, controlling nutritional status.

**Table 3:** Univariate and multivariate analysis for predicting the risk of death in patients with COVID-19

	Univariate			Multivariate	
	OR (95%CI)	*p*		OR (95%CI)	*p*
CONUT score	1.738 (1.494–2.021)	0.000		1.410 (1.089–1.825)	0.009
Age (years)	1.082 (1.055–1.111)	0.000		1.075 (1.031–1.120)	0.001
Gender	0.552 (0.330–0.923)	0.024			
Dyspnea	2.083 (1.119–3.876)	0.021		3.570 (1.157–11.020)	0.027
Hypertension	2.770 (1.655–4.637)	0.000			
CAD	3.914 (2.052–7.466)	0.000		4.858 (1.303–18.118)	0.019
Neutrophils (10^9^/L)	1.508 (1.354–1.679)	0.000			
Platelet (10^9^/L)	0.990 (0.986–0.994)	0.000			
CRP (mg/L)	1.024 (1.018–1.030)	0.000		1.011 (1.002–1.020)	0.012
AST (U/L)	1.032 (1.020–1.045)	0.000			
LDH (U/L)	1.012 (1.009–1.014)	0.000		1.010 (1.007–1.014)	0.000
Acute cardiac injury	24.937 (12.867–48.330)	0.000		4.707 (1.830–12.106)	0.001
Creatinine (µmol/L)	1.016 (1.008–1.024)	0.000			
Total bilirubin	1.073 (1.036–1.110)	0.000			
Blood glucose (mmol/L)	1.136 (1.064–1.214)	0.000			
Antibiotic therapy	9.714 (2.966–31.814)	0.000			
Glucocorticoids	3.498 (2.073–5.902)	0.000			

## Abbreviations

ARDS: acute respiratory distress syndrome
AST: aspartate aminotransferase
AUC: area under curve
CAD: coronary artery disease
CKD: chronic kidney disease
CK-MB: creatine kinase-myocardial isoenzyme
CONUT: controlling nutritional status
COPD: chronic obstructive pulmonary disease
COVID-19: coronavirus disease 2019
CRP: C-reactive protein
cTnI: cardiac troponin I
LDH: lactate dehydrogenase
ROC: receiver operating characteristic
TC: total cholesterol
