# Supplementary material for: Evaluation of the nutritional status in patients with COVID-19
Source: J Clin Biochem Nutr. 2020 Aug 6;67(2):116–21. doi: 10.3164/jcbn.20-91 (PMC7533862; doi:10.3164/jcbn.20-91)
Supplement: Supplemental Figure 1 [file jcbn20-91st01.pdf]

Supplemental Table 1. The evaluation of malnutrition by the CONUT score

	Range and score			
Albumin (g/dl)	≥3.50	3.00–3.49	2.50–2.99	<2.50
Score	0	2	4	6
Total cholesterol (mg/dl)	≥180	140–179	100–139	<100
Score	0	1	2	3
Lymphocyte counts (/mm^3)	≥1,600	1,200–1,599	800–1,199	<800
Score	0	1	2	3

The CONUT score was calculated from serum albumin levels, total cholesterol and lymphocyte counts.
